# Plug-and-play high-frequency feature enhancement for plant image super-resolution

**DOI:** 10.3389/fpls.2025.1723354

**Published:** 2025-11-25

**Authors:** Ling Xu, Bin Qiu, Huijun Xu, Xiaoli Zhu

**Affiliations:** 1 Department of Computer and Information Security Management, Fujian Police College, Fuzhou, China; 2 School of Economics and Management, Tongji University, Shanghai, China; 3 Department of Penalty Execution, Fujian Police College, Fuzhou, China

**Keywords:** plant image super-resolution, high-frequency feature enhancement, deep learning, UAV imagery, phenotyping, crop monitoring

## Abstract

**Introduction:**

High-resolution plant imagery is vital for phenotyping, disease monitoring, and precision agriculture. However, image acquisition in real-world conditions is frequently limited by sensor resolution, cost, and environmental noise, resulting in low-quality images. While deep learning-based super-resolution (SR) approaches show promise, they often fail to recover fine structural details that are essential for plant science applications.

**Methods:**

We propose a plug-and-play high-frequency feature enhancement (HF-FE) module that can be seamlessly integrated into existing SR architectures. The module selectively amplifies high-frequency information, thereby improving the reconstruction of subtle details such as leaf venation, lesion boundaries, and texture patterns, while maintaining computational efficiency. Performance was evaluated on three diverse plant datasets: an oil palm dataset for large-scale plantation imagery, the UAV-based AqUAVPlant dataset for aquatic plants, and the Plant Pathology 2020 dataset for crop disease imagery.

**Results:**

Across all datasets, models incorporating the HF-FE module achieved consistent improvements over state-of-the-art (SOTA) baselines, with notable gains in PSNR and SSIM. Visual assessments further confirmed enhanced clarity of fine structural features, particularly in challenging plant imaging scenarios.

**Discussion:**

The proposed HF-FE module provides a flexible and effective enhancement strategy for plant image SR. By improving the fidelity of reconstructed plant imagery, it supports more accurate visualization and analysis, offering a methodological advancement that contributes to intelligent plant sensing, supports digital agriculture, and facilitates sustainable crop management.

## 1 Introduction

Global challenges such as climate change, population growth, and increasing resource constraints have intensified the demand for efficient and sustainable agricultural practices Schulte et al. (2022). High-quality plant imagery Jiang et al. (2024) underpins many modern solutions in plant science, including precise phenotyping Pieruschka and Schurr (2019), early disease detection Buja et al. (2021), and spatially resolved crop management Raghuram et al. (2022); however, practical image acquisition in field or UAV settings is often limited by sensor resolution, cost, and environmental noise, producing low-resolution (LR) data that hinder accurate analysis Olson and Anderson (2021).

Deep learning–based image SR Xu et al. (2024) has become a powerful approach for reconstructing high-resolution (HR) images from LR inputs, with methods such as EDSR Lim et al. (2017), RCAN Zhang et al. (2018) and more recently transformer-based models Liang et al. (2021); Zhang et al. (2024); Xu et al. (2025) substantially advancing reconstruction fidelity on generic benchmark datasets. These advances have motivated applications of SR in remote sensing Wang et al. (2022) and plant phenotyping Aslahishahri et al. (2021), where spatial detail is critical for downstream biological interpretation Kolhar and Jagtap (2021); Liu et al. (2022).

Most general-purpose SR Cap et al. (2021); Ovečka et al. (2022) architectures are optimized for natural image benchmarks, and they often oversmooth or suppress exactly the high-frequency structures that plant scientists and agronomists care most about. In practice, agronomic decision-making frequently depends on subtle visual cues: vein topology and vein density for phenotyping Huang et al. (2023) and stress assessment Istiak et al. (2024); thin lesion rims, speckle-like infection patterns, and chlorotic edges for early disease diagnosis Zhu et al. (2021); Sathya and Rajalakshmi (2022); Yeswanth et al. (2022); serrated and slender leaf boundaries for species- or health-related morphology in aquatic vegetation; and palm frond geometry and crown contours for stand counting and canopy assessment in plantation monitoring. These biologically meaningful structures are thin, branching, high-frequency patterns that generic SR models tend to degrade. To address this gap, we introduce a plug-and-play High-Frequency Feature Enhancement (HF-FE) module that explicitly targets biologically relevant high-frequency content in plant images. The proposed module combines a Laplacian-based High-Frequency Enhancement Mechanism (HIEM) with a Hybrid Attention (HA) mechanism to (i) extract and amplify fine edge and texture information (e.g., venation, lesion boundaries, palm canopy edges), and (ii) suppress irrelevant background noise. Unlike simply deepening an SR backbone, the HF-FE branch can be inserted into different SR architectures—including CNN-based, attention-based, and Transformer-based models—with minimal modification. As we will show, this design yields consistent gains across diverse plant imaging scenarios, including UAV plantation imagery, aquatic vegetation surveys, and close-range disease monitoring.

We validate the HF-FE module on three representative plant imaging scenarios: (i) oil palm plantation imagery (large-scale remote sensing/UAV data), (ii) the UAV-based AqUAVPlant high-resolution aquatic plant dataset, and (iii) the Plant Pathology 2020 dataset widely used for disease imagery benchmarking. These datasets span diverse imaging scales and conditions, allowing us to assess robustness across practical plant science contexts. Experimental results demonstrate consistent quantitative gains and superior visual recovery of fine structural details compared with strong baselines, indicating the effectiveness of targeted high-frequency enhancement for plant image reconstruction.

By introducing a simple, modular HF-FE component that can be integrated into current SR pipelines, this work contributes a practical tool for improving the visual fidelity of plant imagery. We anticipate that higher-fidelity reconstructions will facilitate more reliable visual analysis in phenotyping and field monitoring, and provide a methodological building block for future integration into end-to-end intelligent plant sensing systems.

## 2 Materials and methods

### 2.1 Datasets and experimental setup

To evaluate the effectiveness and generalizability of the proposed HF-FE module, we conducted experiments on three publicly available plant image datasets that cover diverse ecological and agricultural scenarios: the Oil Palm Dataset Zheng et al. (2021), the AqUAVPlant Dataset Istiak et al. (2024), and the Plant Pathology 2020 Dataset Thapa et al. (2020).

#### 2.1.1 Data acquisition

The Oil Palm dataset provides a collection of high-resolution UAV images of oil palm plantations, primarily collected in Southeast Asia. The dataset was designed to support research on precision agriculture and plantation management by enabling automated monitoring of tree health, canopy size, and plantation density. Images were captured under natural field conditions with variations in illumination, perspective, and background complexity, thus reflecting real-world challenges for plant image analysis. The dataset contains both raw UAV imagery and annotated samples, making it suitable for evaluating reconstruction tasks that require fine-grained structural details.

The AqUAVPlant dataset is a recently published large-scale benchmark for aquatic plant classification and segmentation, acquired using UAV-based high-resolution imaging systems. It comprises images of aquatic environments featuring diverse plant species under dynamic water-surface conditions. The dataset emphasizes fine-grained classification and boundary delineation of aquatic vegetation, thereby offering a unique testing ground for algorithms that must address subtle textural features and complex environmental degradations such as reflections, turbidity, and varying illumination. This dataset is particularly relevant for validating the robustness of SR reconstruction in aquatic agricultural monitoring and environmental conservation contexts.

The Plant Pathology 2020 dataset was introduced as part of the Fine-Grained Visual Categorization (FGVC7) challenge hosted on Kaggle. It contains thousands of leaf images representing healthy plants and multiple disease categories, including complex cases of multiple concurrent infections. The dataset is designed for fine-grained disease recognition in plants, highlighting subtle variations in leaf texture, color, and lesion distribution. Due to its real-world variability and the presence of small, high-frequency pathological features, this dataset provides a rigorous benchmark for assessing the ability of SR methods to recover critical details that directly impact downstream plant health monitoring.

Together, these three datasets encompass a wide range of plant science applications, from plantation-scale monitoring to aquatic vegetation assessment and disease diagnosis. Their diversity in acquisition conditions and target tasks makes them highly suitable for evaluating the effectiveness of the proposed SR reconstruction approach in enhancing fine structural features critical to plant science research.

#### 2.1.2 Data augmentation and preprocessing

For all datasets, we followed a unified preprocessing and evaluation protocol. Each dataset was split into 80% training and 20% testing using a fixed random seed, ensuring reproducibility. From each training HR image, we extracted random 192 × 192 HR patches and applied standard geometric augmentations (random horizontal/vertical flipping and rotations of 0°, 90°, 180°, or 270°) to increase diversity. LR counterparts were then generated from these HR patches using bicubic downsampling with scale factors ×2, ×3, and ×4. Importantly, LR patches were synthesized strictly from the training split; HR test images were never used during training.

All baseline SR models and their enhanced variants with our HF-FE module were trained under identical data splits, augmentations, optimizers, and schedules to ensure a fair comparison. During evaluation, we report PSNR and SSIM on the Y channel for each scale factor, following standard SR practice. Reported scores are averaged over the entire held-out 20% test split of each dataset.

#### 2.1.3 Experimental environment and model training

All experiments were conducted on a high-performance workstation running Ubuntu 24.04 LTS with 128 GB RAM and an Intel Core i9-14900K CPU. Model training and evaluation were accelerated using two NVIDIA GeForce RTX 4090 GPUs, each equipped with 24 GB of memory. The software environment was managed through Conda 25.7.0, with experiments implemented in Python 3.10 and PyTorch 2.2.2. GPU acceleration was enabled using CUDA 13.0 and cuDNN 9.13.0. This configuration ensured sufficient computational resources for training SR models on high-resolution plant image datasets, and all experiments were reproducible within the specified hardware and software environment.

The proposed model was trained end-to-end using paired LR–HR patches. We used the Adam optimizer (*β*_1_ = 0.9*, β*_2_ = 0.999) with an initial learning rate of 1 × 10^−4^. The learning rate was reduced by half every 100,000 iterations to stabilize convergence. We applied gradient clipping with a maximum norm of 5 to prevent exploding gradients. Separate models were trained for ×2, ×3, and ×4 SR. A total of 400,000 training iterations were conducted for each experiment. Here, “iteration” refers to one optimization step on a randomly sampled batch of cropped patches, which is standard practice in patch-based SR training. The *L*_1_ loss function was adopted to minimize pixel-wise differences between reconstructed SR images and their ground-truth HR counterparts, given its superior ability to preserve sharp structural details compared with *L*_2_ loss.

### 2.2 Network setup

**Figure 1:**
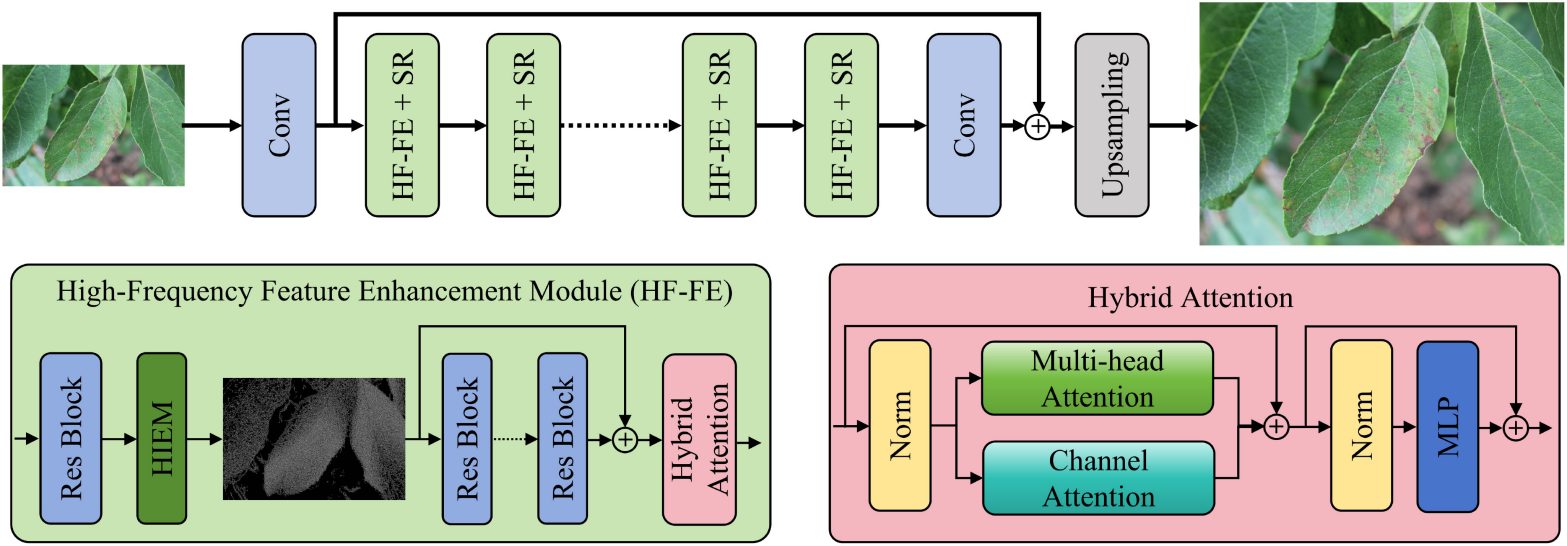
Overview of the proposed HFFEN. The model consists of three parts: (i) a convolutional layer for shallow feature extraction, (ii) multiple HF-FE + SR modules for progressive detail restoration, and (iii) an upsampling module for final HR image reconstruction. Each HF-FE + SR module integrates a standard SR pathway with an HF-FE block that includes a Laplacian-based HIEM and a HA mechanism for spatial and channel-wise feature refinement. This design enables effective recovery of fine structures such as leaf veins and disease spots in plant images.

To enhance the restoration of fine-grained details and texture information in plant images, we propose a HF-FE Network (HFFEN), as shown in **Figure 1**. The network is designed to fully exploit the complementary strengths of conventional SR models and the proposed HF-FE mechanism, which enables more effective recovery of subtle structural patterns—such as veins, edges, and lesion boundaries—often lost during degradation. The overall architecture comprises three main components: (i) shallow feature extraction, (ii) a series of HF-FE + SR modules for progressive enhancement, and (iii) an upsampling module for final HR image reconstruction.

#### 2.2.1 Shallow feature extraction

Given an input LR image $I_{LR}\in {\mathbb{R}}^{H\times W\times C}$, we employ a convolutional layer to extract shallow features:

(1) $$F_{0}=H_{conv}(I_{LR}),$$

where *H_conv_*(·) denotes a convolution operation, and *F*_0_ represents the shallow feature maps. These shallow features primarily contain low-level visual cues such as edges, textures, and color gradients, which are critical for initiating the restoration process. While the convolutional layer efficiently captures local spatial patterns, its receptive field is limited; thus, the subsequent HF-FE + SR modules are responsible for enhancing both the local and global context.

#### 2.2.2 HF-FE + SR

Rather than using a single deep SR network, the proposed HFFEN adopts *N* cascaded modules that combine a baseline SR branch and an HF-FE branch. The process can be expressed as:

(2) $$F_{n}=H_{HF-FE+SR}(F_{n-1}),\;n=1,2,\ldots ,N,$$

where $H_{HF-FE+SR}(\cdot )$ denotes the transformation of one HF-FE + SR block. Each module contains two parallel branches: (i) the SR pathway contributes to the overall image fidelity by refining global spatial patterns and ensuring stable convergence during training, (ii) the HF-FE module is responsible for highlighting high-frequency details through a HIEM, which employs a Laplacian filtering operation to capture edge and texture components from the intermediate feature maps. This operation emphasizes fine-grained structures in plant images—such as disease spots or leaf boundaries—while preserving the semantic consistency of global context.

(3) $$F_{HF}=H_{HF-FE}(F_{n-1}),$$

where $H_{HF-FE}(\cdot )$ denotes a composite Laplacian-based enhancement operator consisting of a fixed Laplacian filter, several residual blocks, and a HA mechanism. It can be expressed as:

(4) $$H_{HF-FE}(x)=HA(R(L*x)),$$

where L is the Laplacian filter, R(·) represents a stack of residual blocks, and *HA*(·) denotes the HA mechanism. This design allows *H_HF_*_−_*_FE_*(·) to enhance high-frequency components while adaptively emphasizing informative spatial and channel-wise cues for reconstruction. The enhanced features from the HF-FE branch and the SR branch are fused by element-wise summation:

(5) $$F_{n}=F_{SR}+F_{HF}.$$

This mechanism allows the model to maintain a balance between the global structural consistency (*F_SR_*) 163 and the local high-frequency enhancement (*F_HF_*).

**Algorithm 1** summarizes the forward path of one HF-FE + SR module. The SR branch focuses on global structural fidelity, while the HF-FE branch applies the Laplacian-based HIEM, followed by residual refinement and a HA mechanism that fuses multi-head self-attention with channel attention. The two branches are fused by element-wise summation, *F_n_*= *F_SR_*+ *F_HF_*. Stacking *N* such modules produces progressively refined feature maps. After the final module, a convolutional aggregation layer produces *F_D_*, which is fused with the shallow feature map *F*_0_, and then upsampled via pixel shuffle to obtain the final super-resolved image *I_SR_*. We use consistent notation for these feature maps throughout (*F_SR_, F_HF_, F_n_, F_D_, I_SR_*) to improve reproducibility.

Algorithm 1

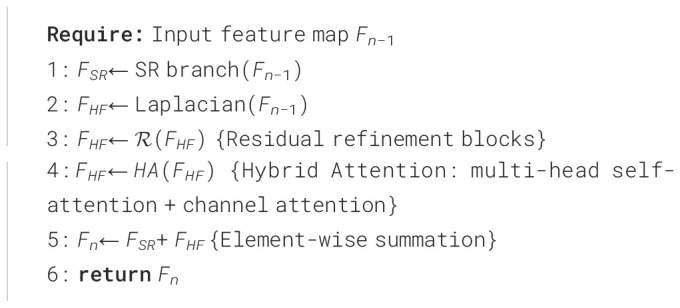

#### 2.2.3 Hybrid attention mechanism

To further improve feature representation, the HF-FE branch incorporates a HA mechanism consisting of multi-head self-attention (MSA) and channel attention (CA). The HA mechanism can be formulated as:

(6) $$F_{HA}=H_{MSA}(F_{in})+H_{CA}(F_{in}).$$

Here, $H_{MSA}(\cdot )$ captures long-range spatial dependencies to improve contextual understanding, while *H_CA_*(·) adjusts channel-wise feature importance based on semantic relevance. Both attention outputs are integrated via normalization and a lightweight MLP layer:

(7) $$F_{out}=H_{MLP}(\text{Norm}(F_{HA})).$$

This design ensures stable training and effective fusion of multi-dimensional dependencies, significantly enhancing detail recovery in complex plant textures.

#### 2.2.4 Progressive enhancement and reconstruction

Multiple HF-FE + SR modules are sequentially stacked to form a progressive enhancement pathway. Through this hierarchical structure, features are continuously refined:

(8) $$F_{D}=H_{conv}(F_{N}),$$

where *H_conv_*(·) represents the final convolution layer for feature aggregation, and *F_N_*is the last enhanced 184 feature map. The final HR image *I_SR_* is generated via an upsampling module using pixel-shuffle:

(9) $$I_{SR}=H_{ups}(F_{D}+F_{0}).$$

Here, *H_ups_*(·) denotes the upsampling operation. By fusing both shallow and deep features, the model achieves a synergistic balance between fine-grained detail preservation and global structural coherence, enabling the restoration of clearer and more natural plant images. Our model is formally defined by **Equations 1**–**9**.

The proposed HFFEN seamlessly integrates conventional SR models with a high-frequency enhancement pathway and a HA mechanism. This design enables the model to (1) extract and preserve critical high-frequency information via Laplacian-based filtering, (2) enhance multi-level feature interactions through attention fusion, and (3) progressively refine both local and global details through sequential HF-FE + SR modules. As a result, the network achieves superior fidelity and realism in reconstructed images, which benefits downstream plant analysis tasks such as disease diagnosis and phenotyping.

## 3 Results

### 3.1 Comparison experiments

To comprehensively evaluate the performance of the proposed model, we conducted experiments on three benchmark plant image datasets. We compared our method against several SOTA SR algorithms, including EDSRLim et al. (2017), RCANZhang et al. (2018), HANNiu et al. (2020) and SwinIRLiang et al. (2021). All models were trained and tested under the same experimental conditions for fair comparison.

#### 3.1.1 Quantitative comparison

**Table 1** presents the quantitative comparison results of the proposed method and several SOTA SR algorithms, under upscaling factors of ×2, ×3, and ×4. The proposed HF-FE module consistently improves reconstruction quality when integrated with different SR backbones.

**Table 1:** Quantitative comparison (PSNR/SSIM) of different SR methods on three plant datasets under upscaling factors ×2, ×3, and ×4.

Methods	Scale	Oil Palm		AqUAVPlant		Plant Pathology 2020	
		PSNR	SSIM	PSNR	SSIM	PSNR	SSIM
EDSR	×2	37.40	0.9561	35.32	0.9390	38.05	0.9532
EDSR+Our	×2	**37.76**	**0.9640**	**35.63**	**0.9475**	**38.30**	**0.9584**
RCAN	×2	37.64	0.9596	35.60	0.9460	38.19	0.9564
RCAN+Our	×2	**37.98**	**0.9637**	**35.81**	**0.9537**	**38.37**	**0.9590**
HAN	×2	37.86	0.9636	35.70	0.9487	38.34	0.9579
HAN+Our	×2	**38.18**	**0.9704**	**35.94**	**0.9549**	**38.49**	**0.9598**
SwinIR	×2	38.23	0.9701	36.10	0.9560	38.55	0.9607
SwinIR+Our	×2	**38.38**	**0.9749**	**36.26**	**0.9595**	**38.64**	**0.9619**
EDSR	×3	33.55	0.9141	32.05	0.9018	34.52	0.9231
EDSR+Our	×3	**33.89**	**0.9237**	**32.41**	**0.9107**	**34.89**	**0.9314**
RCAN	×3	33.80	0.9180	32.20	0.9051	34.75	0.9304
RCAN+Our	×3	**34.11**	**0.9251**	**32.47**	**0.9122**	**35.05**	**0.9364**
HAN	×3	33.90	0.9201	32.31	0.9069	34.80	0.9314
HAN+Our	×3	**34.21**	**0.9261**	**32.58**	**0.9128**	**35.10**	**0.9385**
SwinIR	×3	34.40	0.9271	32.58	0.9156	35.12	0.9405
SwinIR+Our	×3	**34.56**	**0.9301**	**32.69**	**0.9190**	**35.27**	**0.9435**
EDSR	×4	30.10	0.8721	29.78	0.8616	32.10	0.8839
EDSR+Our	×4	**30.38**	**0.8805**	**30.15**	**0.8717**	**32.42**	**0.8910**
RCAN	×4	30.24	0.8754	29.91	0.8649	32.25	0.8878
RCAN+Our	×4	**30.45**	**0.8820**	**30.22**	**0.8759**	**32.56**	**0.8930**
HAN	×4	30.33	0.8776	29.96	0.8669	32.30	0.8880
HAN+Our	×4	**30.49**	**0.8826**	**30.27**	**0.8739**	**32.57**	**0.8941**
SwinIR	×4	30.54	0.8855	30.24	0.8749	32.75	0.8980
SwinIR+Our	×4	**30.63**	**0.8865**	**30.35**	**0.8787**	**32.87**	**0.9008**

The best results are highlighted in bold.

For the ×2 upscaling, our method achieves the highest PSNR and SSIM across all datasets. For instance, when added to EDSR, PSNR improves from 37.40 dB to 37.76 dB (+0.36 dB) and SSIM from 0.9561 to 0.9640 on the Oil Palm dataset. Similarly, RCAN+Our achieves 37.98 dB/0.9637, and HAN+Our reaches 38.18 dB/0.9704, surpassing their respective baselines by 0.2–0.4 dB. The most advanced baseline, SwinIR, also benefits from our module, improving from 38.23 dB to 38.38 dB (+0.15 dB) on Oil Palm and from 38.55 dB to 38.64 dB (+0.09 dB) on Plant Pathology 2020. These results indicate that our enhancement block provides complementary gains even for transformer-based SR architectures.

Under the $*times*3 upscaling setting, similar performance trends are observed. EDSR+Our attains 33.89 dB/0.9237 on Oil Palm, improving by 0.34 dB and 0.0086 in SSIM compared to the baseline. RCAN+Our further raises PSNR to 34.11 dB and SSIM to 0.9251, while HAN+Our achieves 34.21 dB/0.9261, both surpassing their counterparts by 0.3–0.4 dB. On the challenging AqUAVPlant dataset, the proposed module enhances texture recovery and structural consistency, as seen in RCAN+Our (32.47 dB/0.9122) and HAN+Our (32.58 dB/0.9128). SwinIR+Our again obtains the best overall results (34.56 dB/0.9301 on Oil Palm and 35.27 dB/0.9435 on Plant Pathology 2020), confirming the robustness and scalability of our design.

At the ×4 scale, which presents the greatest reconstruction challenge, our method still demonstrates consistent improvements. The EDSR baseline increases from 30.10 dB to 30.38 dB on Oil Palm and from 32.10 dB to 32.42 dB on Plant Pathology 2020. RCAN+Our achieves 30.45 dB/0.8820, outperforming RCAN by 0.21 dB and 0.0071 SSIM. Likewise, HAN+Our reaches 30.49 dB/0.8826 on Oil Palm and 32.57 dB/0.8941 on Plant Pathology 2020, while SwinIR+Our delivers the best overall result of 30.63 dB/0.8865 and 32.87 dB/0.9008, respectively.

Overall, the proposed HF-FE module yields consistent PSNR and SSIM improvements ranging from +0.1 dB to +0.4 dB across all scales and datasets. The enhancement effect is more pronounced for earlier CNN-based architectures such as EDSR and RCAN, while transformer-based models (e.g., SwinIR) still gain moderate yet stable performance boosts. This demonstrates that our module effectively complements diverse SR backbones by refining high-frequency representations and improving structural fidelity in plant image reconstruction.

#### 3.1.2 Qualitative comparison

**Figure 2:**
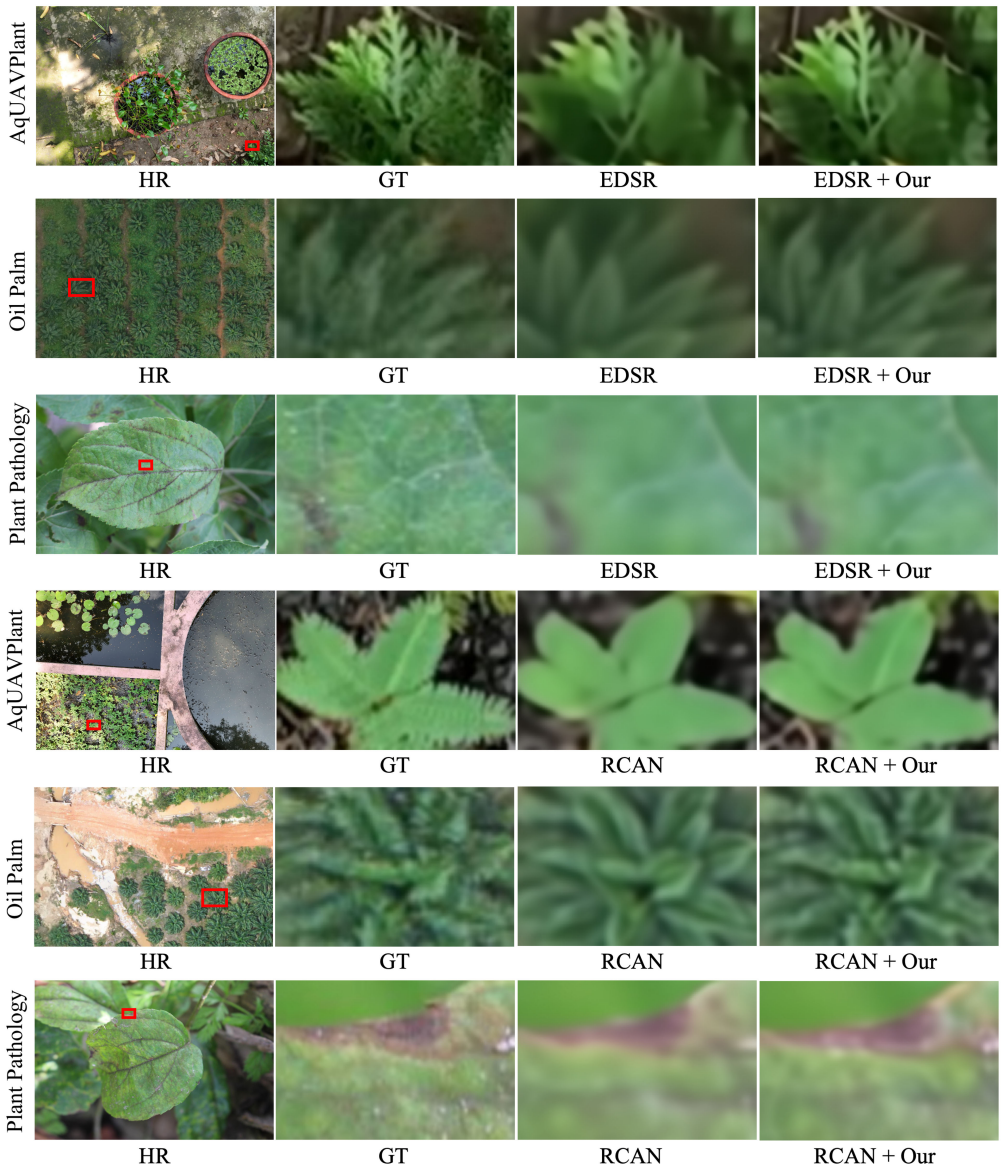
Visual comparison of reconstruction results between EDSR/RCAN and their enhanced versions (EDSR+Our, RCAN+Our) on the AqUAVPlant, Oil Palm, and Plant Pathology 2020 datasets under ×4 upscaling. The proposed HF-FE module recovers sharper serrated leaf boundaries and slender aquatic plant contours in AqUAVPlant, improves palm frond edge definition and canopy structure in Oil Palm imagery, and enhances lesion rims and fine venation patterns in Plant Pathology 2020. These fine structures are agronomically meaningful and are often blurred or oversmoothed in the baseline outputs.

**Figure 3:**
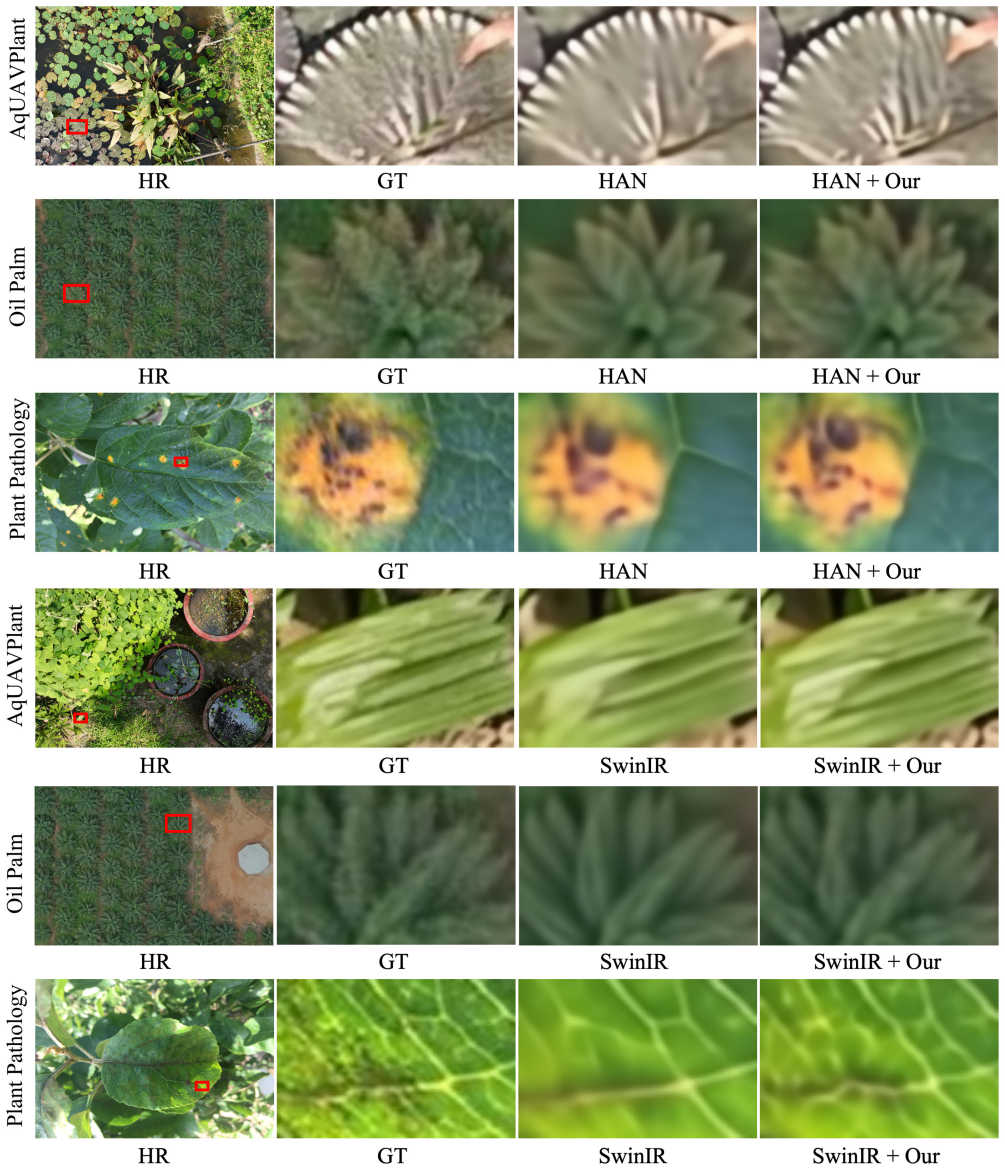
Visual comparison of reconstruction results between HAN/SwinIR and their enhanced versions (HAN+Our, SwinIR+Our) on the AqUAVPlant, Oil Palm, and Plant Pathology 2020 datasets under ×4 upscaling. The enhanced models better preserve high-frequency plant structures, reduce oversmoothing, and maintain more coherent texture continuity across leaf surfaces. In particular, lesion boundaries and vein patterns become more distinct, and palm canopy edges remain more stable, which supports downstream tasks such as health monitoring and stress assessment.

To further assess the visual improvement brought by our proposed high-frequency feature enhancement module, a qualitative comparison was conducted on three representative plant datasets: AqUAVPlant, Oil Palm, and Plant Pathology 2020, as illustrated in **Figures 2**, **3**. Compared with their respective baselines (EDSR, RCAN, HAN, and SwinIR), our enhanced models demonstrate clear advantages in visual fidelity, particularly in regions containing fine leaf structures, stem textures, and disease spots. In the AqUAVPlant dataset, our method effectively restores the serrated edges and slender leaf contours that are heavily blurred in the original reconstructions. For the Oil Palm dataset, the improved models produce sharper palm fronds and maintain a more consistent texture distribution, avoiding the over-smoothing effect observed in the baseline methods. In the Plant Pathology 2020 dataset, our approach significantly enhances the visibility of lesion boundaries and venation details, leading to more accurate visual cues for potential disease identification. Overall, these results confirm that the integration of the proposed enhancement module with existing SR architectures consistently improves perceptual sharpness and texture realism, while maintaining stable color consistency across different plant species and imaging conditions.

### 3.2 Ablation study

To further validate the effectiveness of each component in the proposed model, we conducted ablation experiments focusing on the HIEM and the HA mechanism. **Table 2** summarizes the quantitative results on the Oil Palm, AqUAVPlant, and Plant Pathology 2020 datasets under the ×2 upscaling factor. The baseline model is SwinIR, and all variants were trained under identical settings for fair comparison.

**Table 2:** Ablation study on the effectiveness of the HIEM and HA mechanisms on three datasets under ×2 upscaling.

Model Variant	Oil Palm		AqUAV Plant		Plant Pathology 2020		Time (s)	FLOPs (G)
	PSNR	SSIM	PSNR	SSIM	PSNR	SSIM		
Baseline (SwinIR)	38.23	0.9701	36.10	0.9560	38.55	0.9607	0.152	243.7
+HIEM	38.32	0.9735	36.20	0.9581	38.62	0.9612	0.158	249.3
+HA	38.28	0.9729	36.14	0.9571	38.58	0.9610	0.165	256.9
+HIEM + HA	**38.38**	**0.9749**	**36.26**	**0.9595**	**38.64**	**0.9619**	0.169	260.9

PSNR/SSIM are reported on the Y channel. Time is measured in seconds per image (s/img) on our workstation. FLOPs are theoretical multiply-add operations for a 192 × 192 input patch. Best results are highlighted in bold.

Compared with the baseline, incorporating the HIEM module consistently improves the PSNR and SSIM values across all three datasets (e.g., from 38.23 dB/0.9701 to 38.32 dB/0.9735 on Oil Palm). This improvement demonstrates that the HIEM effectively enhances fine-grained edge textures and high-frequency structures, leading to sharper and more realistic plant details. The addition of the HA mechanism also brings steady gains in reconstruction accuracy, highlighting its ability to capture complementary global–local contextual dependencies and refine feature representations. When both modules are combined, the full model achieves the best overall performance, yielding PSNR = 38.38 dB and SSIM = 0.9749 on the Oil Palm dataset, with similar improvements observed on AqUAVPlant and Plant Pathology 2020.

Adding the full HF-FE branch (HIEM + HA) increases total parameter count and theoretical FLOPs by less than 10%. On our workstation, the average inference time rises from 0.152 s/img to 0.169 s/img (i.e., an overhead of ∼0.017 s/img). Thus, the HIEM and HA components yield measurable gains in the recovery of high-frequency plant structures with only modest runtime and complexity overhead. We note that these timing results are measured on high-end desktop GPUs; future work will profile lightweight variants of the HF-FE module on embedded computer platforms and UAV-mounted hardware to assess in-field, near-real-time feasibility for precision agriculture and ecological monitoring.

These results confirm that both HIEM and HA contribute complementary benefits: HIEM emphasizes the recovery of structural and textural high-frequency details, while HA enhances semantic consistency and inter-channel feature relevance. Together, they form a synergistic mechanism that significantly improves overall reconstruction quality in complex plant imaging scenarios.

## 4 Discussion

### 4.1 Overview of key findings

In this study, we proposed a plug-and-play HF-FE module and its associated HFFEN, which integrate a HIEM and a HA mechanism to improve the quality of plant image SR. Experimental results on three representative plant datasets demonstrate that the proposed method consistently surpasses several SOTA SR models, including EDSR, RCAN, HAN, and SwinIR, across multiple upscaling factors. The improvements are evident not only in quantitative performance but also in visual fidelity, particularly in restoring fine textures such as leaf veins, petiole boundaries, and disease-affected regions. These results confirm that high-frequency information plays a crucial role in recovering fine plant details, which are often essential for subsequent analysis tasks such as disease diagnosis, phenotyping, and morphological assessment.

### 4.2 Comparison with prior research

Traditional CNN-based SR models (e.g., EDSR, RCAN) mainly focus on deepening network layers or enlarging receptive fields to enhance representation capacity. While these strategies improve global structural restoration, they tend to smooth out the high-frequency information that characterizes delicate biological textures. More recent Transformer-based methods (e.g., SwinIR) have demonstrated stronger global modeling capabilities but still lack an explicit mechanism to emphasize high-frequency regions critical to plant imagery. Our method distinguishes itself by introducing a frequency-domain enhancement mechanism—the HIEM—that extracts edge and texture information via Laplacian filtering and residual aggregation. This design bridges the gap between frequency-based enhancement and spatial feature modeling, effectively combining the interpretability of classical image processing with the adaptability of deep learning. Meanwhile, the HA mechanism complements this process by adaptively recalibrating spatial and channel dependencies, ensuring that important visual cues such as lesion textures or canopy contours are emphasized while suppressing redundant background information. Together, these mechanisms allow our model to achieve a balance between local detail recovery and global structural consistency, advancing beyond the performance of existing SOTA models.

### 4.3 Limitations and potential improvements

Despite the promising results, several limitations should be acknowledged. First, although the added computational cost of the HF-FE module is modest on a workstation GPU, deploying the full model on embedded, low-power hardware such as UAV-mounted processors or handheld scouting devices remains non-trivial. Developing lighter-weight or compressed variants of the HF-FE branch is therefore a key direction for enabling in-field and near-real-time use in precision agriculture.

Second, in this study all LR inputs were generated via bicubic downsampling of HR patches. This follows standard SR practice, but it does not fully reproduce the degradations that occur in real agricultural and ecological monitoring scenarios, such as UAV motion blur, sensor noise, canopy shadows, low illumination, or surface reflections in aquatic vegetation scenes. A natural extension of this work is to incorporate degradation-aware or domain-adaptive training that explicitly models these non-ideal capture conditions.

Third, the current formulation operates on single RGB images. Many real-world plant monitoring pipelines are temporal (e.g., UAV flyovers, time-lapse stress monitoring) or multimodal (e.g., RGB + multispectral/hyperspectral sensing). Extending HF-FE to handle temporal consistency and multi-sensor fusion is an important future step.

### 4.4 Integration into the current understanding

The proposed HF-FE framework offers a new perspective on plant image SR by explicitly linking high-frequency information processing with attention-driven context modeling. This approach aligns with a growing recognition in plant phenotyping and precision agriculture that fine-grained visual cues—such as vein density, stomatal patterns, and surface texture—contain critical physiological and diagnostic information. By restoring such details more accurately, our method supports a more reliable extraction of morphological and pathological indicators from high-resolution reconstructions. Furthermore, the plug-and-play nature of our design allows it to be easily integrated into various vision-based agricultural applications, from disease detection networks to spectral reconstruction models, thereby extending its practical relevance beyond image enhancement alone.

In practical terms, the improved reconstruction of palm frond boundaries and crown contours (Oil Palm) supports plantation-scale tasks such as stand counting and canopy health assessment. The sharper delineation of aquatic vegetation boundaries (AqUAVPlant) is directly relevant to habitat mapping and invasive species monitoring in aquatic systems. The enhanced visibility of lesion rims and fine venation patterns (Plant Pathology 2020) provides clearer visual evidence for early disease scouting and stress diagnosis in crop protection. These are core workflows in precision agriculture and intelligent phytoprotection.

Finally, we note that classical frequency-domain SR and edge-enhancement strategies typically apply fixed high-pass or sharpening filters. By contrast, our High-Frequency Enhancement Mechanism (HIEM) explicitly extracts Laplacian-based high-frequency responses and then feeds them through residual refinement and a Hybrid Attention (HA) unit that jointly models spatial (multi-head self-attention) and channel-wise relevance. This learnable, attention-guided treatment of high-frequency cues enables selective preservation of botanically meaningful structures while suppressing irrelevant background texture.

### 4.5 Future research directions

Future work will focus on extending the proposed HF-FE framework along three directions. First, temporal modeling could be introduced to handle video-based plant monitoring, ensuring consistent enhancement across sequential frames. Second, multimodal fusion of RGB, multispectral, and hyperspectral data may further improve the reconstruction of physiologically informative features that are invisible in the visible spectrum. Third, lightweight and adaptive versions of the HF-FE module could be designed to facilitate deployment on edge computing devices and UAV platforms for real-time agricultural monitoring. Additionally, integrating interpretable frequency-domain visualization tools could help better understand how the proposed modules capture and utilize plant-specific textures, fostering more transparent AI-driven plant analysis systems.

In addition, many real agronomic sensing pipelines operate in video mode (e.g., UAV flyovers) or rely on multispectral and hyperspectral measurements for physiological assessment. Extending the HF-FE framework to handle temporal consistency across frames and to fuse complementary spectral modalities is a natural next step toward deployable, intelligent plant monitoring systems.

### 4.6 Summary

Overall, this study presents a robust and generalizable solution to enhance plant image SR through frequency-domain feature amplification and HA optimization. The proposed method not only achieves SOTA performance in reconstruction accuracy but also offers valuable insights into how high-frequency visual cues contribute to the accurate restoration of plant structures. These findings provide a strong foundation for future research toward high-resolution, data-driven plant observation and intelligent agricultural systems.

## Data Availability

The original contributions presented in the study are included in the article/supplementary material. Further inquiries can be directed to the corresponding author.
